# The San Carlo Colossus: An Insight into the Mild Galvanic Coupling between Wrought Iron and Copper

**DOI:** 10.3390/ma16052072

**Published:** 2023-03-03

**Authors:** Chiara Petiti, Carla Martini, Cristina Chiavari, Silvia Vettori, Jean Marie Welter, Paulina Guzmán García Lascurain, Sara Goidanich

**Affiliations:** 1 Department of Chemistry, Materials and Chemical Engineering “Giulio Natta”, Politecnico di Milano, Piazza Leonardo da Vinci, 32, 20133 Milano, Italy; 2 Department of Industrial Engineering, University of Bologna, Viale del Risorgimento, 4, 40136 Bologna, Italy; 3 Department of Cultural Heritage, University of Bologna, Via degli Ariani, 1, 48121 Ravenna, Italy; 4 Institute of Heritage Science-National Research Council (ISPC-CNR), Via Madonna del Piano, 10, 50019 Sesto Fiorentino, Italy; 5 Independent Researcher, 1361 Luxembourg, Luxembourg

**Keywords:** galvanic coupling, wrought iron, copper, historic iron

## Abstract

The San Carlo Colossus, known as *San Carlone*, is a monument constituted by an internal stone pillar support to which a wrought iron structure is attached. Embossed copper sheets are fixed to the iron structure to give the final shape to the monument. After more than 300 years of outdoor exposure, this statue represents an opportunity for an in-depth investigation of long-term galvanic coupling between wrought iron and copper. Most iron elements of the *San Carlone* appeared in good conservation conditions with scarce evidence of galvanic corrosion. In some cases, the same iron bars presented some portions in good conservation conditions and other nearby portions with active corrosion. The aim of the present study was to investigate the possible factors correlated with such mild galvanic corrosion of wrought iron elements despite the widespread direct contact with copper for more than 300 years. Optical and electronic microscopy and compositional analyses were carried out on representative samples. Furthermore, polarisation resistance measurements were performed both on-site and in a laboratory. The results revealed that the iron bulk composition showed a ferritic microstructure with coarse grains. On the other hand, the surface corrosion products were mainly composed of goethite and lepidocrocite. Electrochemical analyses showed good corrosion resistance of both the bulk and surface of the wrought iron, and galvanic corrosion is not occurring probably due to the iron’s relatively noble corrosion potential. The few areas where iron corrosion was observed are apparently related to environmental factors, such as the presence of thick deposits and to the presence of hygroscopic deposits that create localized microclimatic conditions on the surface of the monument.

## 1. Introduction

**Figure 1:**
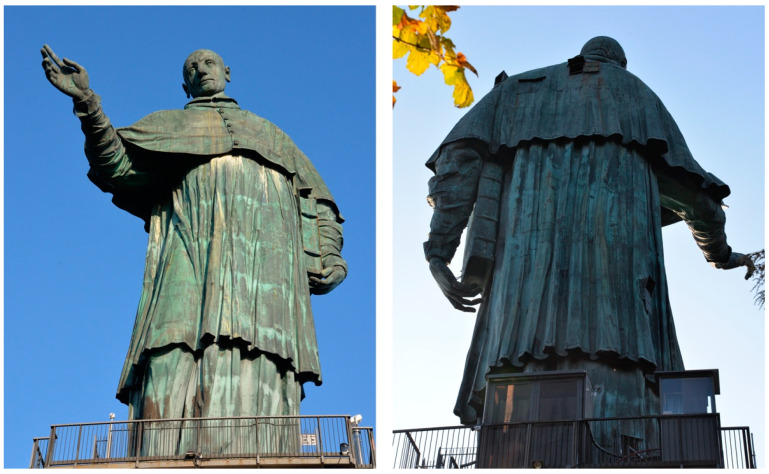
The Colossus of San Carlo Borromeo (1538–1584), Arona (Italy), called *San Carlone*.

**Figure 2:**
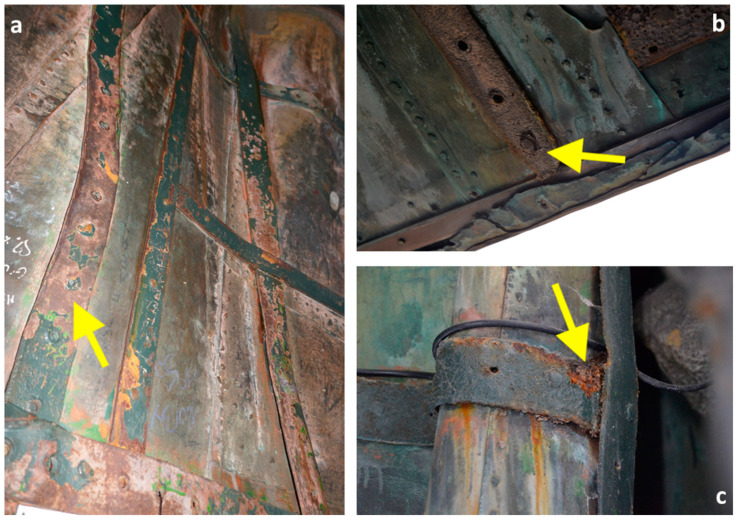
Examples of degradation patterns on iron elements. (**a**) Iron bars in good conservation conditions despite the degradation of the protective coating; (**b**,**c**) small areas characterised by severe corrosion surrounded by almost intact zones.

The Colossus of San Carlo Borromeo (1538–1584), called *San Carlone*, i.e., *Big Saint Charles* (Figure 1) due to its large size, located in Arona on the Lago Maggiore (Piedmont, Italy), was built between 1614 and 1698. The 22 m high statue (which is positioned on an 11 m high granite pedestal) was manufactured by a peculiar construction technique: An internal stone pillar supports a wrought iron structure, embossed with fixed copper sheets. The iron structure, in direct contact with the copper sheets, provides structural support and shapes the statue. Due to its construction technique and more than 300 years of outdoor exposure, the statue represents an opportunity for an in-depth investigation of the long-term galvanic coupling between the wrought iron and copper. Despite the frequent direct contact between the two metals, wrought iron presents only a limited number of areas heavily affected by corrosion, while the rest looks in quite good conservation condition. It has been reported that in a previous restoration, a protective coating was applied [1,2,3]. However, this coating is nowadays heavily deteriorated with large portions of the iron surfaces directly exposed to the atmosphere. The *San Carlone* statue, therefore, should be prone to galvanic corrosion. However, upon visual inspection of the monument, most iron elements of the Colossus appeared in quite good conservation condition despite the general degradation of the protective coating (Figure 2a) with scarce evidence of galvanic corrosion. In contrast, active corrosion of the iron elements was observed regardless of their direct contact with the copper sheets. In some cases, the same iron bars presented some portions in good conservation conditions and other nearby portions with active corrosion (Figure 2b,c). 

The aim of the present study is therefore to identify the factors responsible for the mild galvanic corrosion of the wrought iron elements despite the widespread direct contact with copper for more than 300 years.

Galvanic corrosion is a specific form of corrosion that occurs between two dissimilar metals that are in contact in the presence of a conductive electrolyte [4]. The driving force for this corrosion process is represented by the difference in free corrosion potential between the two metals: The less noble metal acts as the anode and undergoes oxidation, while the nobler one is the cathode and is protected from corrosion [4,5,6]. A notorious case in which corrosion by galvanic coupling has been a conservation issue is the Statue of Liberty in the harbour of New York. The statue was built with an internal structure of iron elements on which an external copper “skin” was joined, giving shape to the female figure of Liberty. In fact, the Statue of Liberty has been previously compared to the *San Carlone* since they present relevant similarities in terms of materials and manufacturing technologies [1]. Due to the higher nobility of copper with respect to iron, severe corrosion of the iron elements of the Statue of Liberty was observed [7]. In this case, galvanic corrosion has been favoured and accelerated by New York’s humid and chlorine-rich environment. 

To understand the galvanic corrosion of iron and its behaviour in different contexts, it is important to know the level of protection provided by the corrosion layers that can form under different exposure conditions. Depending on a series of environmental factors (e.g., oxygen availability, pH, ion concentration of the electrolyte, etc.), several forms of oxides and iron compounds are produced during Fe oxidation, forming a layer commonly referred to as “patina”. A detailed model for the description of the corrosion mechanism of iron was proposed by Evans and Taylor [8] and demonstrated by Stratmann and Streckel [9,10]. Iron corrosion products are often porous, poorly adherent, and often show cracks in most external layers. Therefore, they are not effective in hindering the access of water and oxygen to the metallic surface and in lowering the corrosion rate. In the initial stages of corrosion, a thin oxide/hydroxide film (typically 1–4 nm) is formed with a passivating effect in nonaggressive conditions [11]. During the intermediate stages of corrosion, the formation of two types of the so-called “green rusts” is observed, namely, “green rust I” Fe^II^_2_Fe^III^O_x_(OH)_y_ and “green rust II” Fe^II^Fe^III^O_x_(OH)_y_ [11]. In the final stages of corrosion, the corrosion layers of surfaces exposed to atmospheric corrosion mainly consist of iron oxides and oxides–hydroxides. These oxides are characterized by different levels of crystallinity: In particular, several polymorphs of FeOOH can be present [11,12,13]. 

In general, it was observed that lepidocrocite (γ-FeOOH) is the main phase in the first weeks after the transformation of the green rust, while upon longer exposures the predominant phase becomes goethite (α-FeOOH), as it is the most stable iron oxide–hydroxide. In association with goethite, magnetite (Fe_3_O_4_) can also be found. In fact, its development is promoted when the supply of oxygen is limited, and a slight variation in its availability can favour the stability of one or another of these two compounds [13]. In the case of high concentrations of chlorides in the atmosphere, high relative humidity and low pH, the formation of akaganeite (β-FeOOH) is promoted. In addition, the formation of akaganeite is typical in the proximity of coastal areas or in pits. In these cases, β-Fe_2_(OH)_3_Cl is the typical intermediate product that can be detected in association with green rusts [13]. Moreover, hematite (α-Fe_2_O_3_) and maghemite (γ-Fe_2_O_3_) can be detected among corrosion products. Even if they are seldom produced upon atmospheric corrosion, they have been identified in several cases of cultural heritage artefacts [13]. In fact, hematite occurs in high-temperature corrosion or as transformation products when other corrosion products are heated. Therefore, its presence on artistic or archaeological surfaces can be due to high-temperature treatments performed in the past [13,14]. Maghemite was, instead, identified in archaeological artefacts and on historical iron in the very first phases of atmospheric corrosion [14]. In addition, low crystallinity phases can be detected among the corrosion products, especially in the most internal layer of rust. These amorphous phases are usually constituted by feroxyhyte (δ-FeOOH) and ferrihydrite (Fe^3+^)_2_O_3_·0.5H_2_O [15,16,17,18]. 

The composition of corrosion products can be influenced by the presence and concentration of atmospheric pollutants. When sulphur dioxide (SO_2_) is present, it can influence the corrosion rate of iron. When a significant concentration of SO_2_ is present in the environment, the formation of H_2_SO_4_ in the electrolyte film is promoted with a consequent decrease in its pH. Moreover, sulphuric acid can dissolve oxides to produce FeSO_4_. FeSO_4_ is soluble and hygroscopic, thus favouring the formation of an electrolyte film even with low RH values. Even if the iron patinas resulting from atmospheric corrosion are normally constituted by iron oxides and hydroxides, hydrated phases of FeSO_4_ have also been identified in atmospheric rusts on low alloy steels [19,20]. Moreover, chlorides can play an important role in the atmospheric corrosion of iron-based alloys. Chlorides can promote the formation of akaganeite instead of goethite in the final stages of corrosion. Additionally, chloride ions have a high transport number in water, thus moving easily in the electrolyte film. Thus, when the concentration of chloride ions is significant, an increase in the corrosion rate can be expected [11]. A critical aspect of the atmospheric corrosion of iron elements is connected with the high increase in volume that may be associated with the formation of corrosion products. A notable case of atmospheric corrosion is the one of Ponte di San Michele (St. Michael’s bridge), located in Italy between Paderno d’Adda and Calusco d’Adda and built-in 1889. In this case, the microclimatic conditions to which the iron is exposed and the disposition and interconnection of the different iron elements resulted in corrosion at specific locations with highly expansive effects. This peculiar situation induced significant deformation of many elements of the bridge [21,22]. Besides the qualitative identification of the corrosion products, the quantification of their phases has also been reported in the literature as relevant for understanding the level of protectiveness provided by corrosion layers [15,18,23,24,25]. In particular, some authors suggested calculating a protective ability index (PAI) for the iron patinas based on the ratio between the number of stable phases and reactive ones [26]. The PAI was suggested by Yamashita et al. [26] for the first time. They quantified the amount of goethite (α-FeOOH), i.e., the stable phase, and of lepidocrocite (γ-FeOOH), i.e., the reactive one. Thus, the PAI was defined as follows:

$$\mathrm{PAI}=\frac{\alpha }{\gamma }$$

where α indicates the mass fraction of α-FeOOH and γ the mass fraction of γ-FeOOH. They demonstrated that the PAI is strongly correlated to the corrosion rate of the surface. When α/γ is higher than 1, the rust layer appears quite stable and protective [14]. 

According to European standards and several studies, copper alloys have good resistance to corrosion in moderately aggressive environments [27,28]. In particular, the copper shows lower corrosion rates with respect to iron and carbon steel in the same environments [27]. Furthermore, copper alloys show different corrosion resistances with respect to pure metal thanks to the contribution of alloying elements [28,29,30,31,32]. Therefore, copper and copper alloys have been widely employed in the artistic and architectural fields, especially to produce artefacts and architectural elements for outdoor exposure [28,29,33,34].

## 2. Materials and Methods

### 2.1. Materials

The *San Carlone* underwent an extensive diagnostic campaign. External and internal representative areas at different heights considering both copper and iron elements were investigated by nondestructive in situ methodologies and by laboratory analyses on microsamples. Three samples of iron fragments were taken due to the peculiar function and geometry of the iron elements in the statue. They have been selected, as they were representative of the different conservation conditions observed:

- Sample 44: metallic fragment with slight corrosion despite direct contact with copper collected from the internal part of the chin of the statue.
- Sample 46: metallic fragment not in contact with any copper element and with very slight atmospheric corrosion.
- Sample 87: metallic fragment collected from an area in contact with a copper element showing severe corrosion.
- Sample A: reference sample of 19th-century puddled wrought iron.

In addition, powder samples were taken from iron bars with active corrosion and incoherent, scarcely adherent corrosion products.

### 2.2. Environmental Data

Temperature and relative humidity data collected during 2018 and 2019 were reported and discussed in previous work [1]. The collected data showed that different parts of the statue may present different times of wetness (TOW) as consequence of different solar radiation and ventilation. Based on the BS EN ISO 9223:2012 standard [27], the internal area of the *San Carlone* should be classified between τ3 and τ4.

Annual pollutant concentrations from two monitoring stations located near Arona (Table 1) were downloaded from the regional environmental protection agency (ARPA) website (https://aria.ambiente.piemonte.it/#/qualita-aria/dati, accessed on 20 December 2022). These data show that the area is not characterised by high levels of pollutants compared to urban areas. 

### 2.3. Methods

The composition of iron alloys has been analysed by GDOES and the elemental composition of the inclusions by SEM-EDX. The chemical composition of the corrosion layers has been investigated by means of micro-Raman spectroscopy and XRD. Sample microstructure has been analysed by optical microscopy and SEM, and the corrosion behaviour has been studied by means of electrochemical analysis (corrosion potential, LPR and EIS measurements).

Optical microscope observations were performed with a Leica M205C microscope equipped with a Leica DFC 290 camera. 

SEM-EDX (Zeiss, Jena, Germany) was performed with an ESEM Zeiss EVO 50 EP in extended pressure equipped with an Oxford INCA Energy 200-Pentafet LZ4 spectrometer. The FTIR analysis was carried out by a Thermo Nicolet 6700 spectrophotometer (Thermo Fisher Scientific Inc, Waltham, MA, USA) employing a DTGS detector with a detection range between 4000 and 400 cm^−1^. The XRD analysis was performed with a PANalytical diffractometer X’Pert PRO with radiation CuKα1 = 0.154 nm, operating at 40 kV and 30 mA, investigated range 2θ 3–70°, equipped with X’Celerator multidetector. 

**Figure 3:**
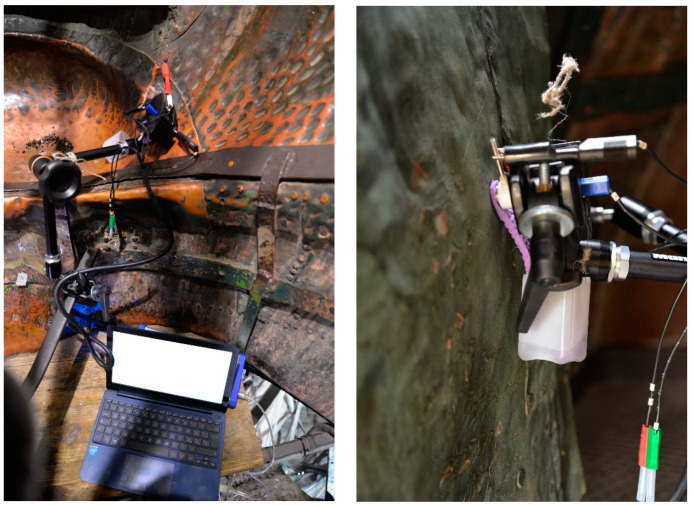
In situ electrochemical measurements with the contact probe.

LPR and EIS measurements have been carried out on-site (Figure 3) by employing the Contact Probe proposed by Letardi [35]. The Contact Probe is constituted by an AISI316L stainless steel counter (CE) and pseudoreference (RE) electrodes embedded in a PTFE case. On polished cross-section of microsamples collected from the same monument, LPR and EIS measurements have been carried out with the Minicell by Amel s.r.l. The Minicell is constituted by a platinum counter electrode and an Ag/AgCl reference electrode hosted in a cylindrical plastic case where the electrolyte flows continuously with a minipump. All LPR, EIS, and E_corr_ measurements were performed with a portable potentiostat Ivium Technologies with CompactStat Ivium® software using oligomineral water (pH around 8 and conductivity around 200 μS/cm) as an electrolyte. 

LPR measurements were performed after 10 min of monitoring time (MT) of the open circuit potential (OCP). The potential ranged ±10 mV with respect to the measured E_corr_ with scan rate of 10 mV/min. EIS measurements were performed after 5 min of monitoring time (MT) and 5 min of stabilization of the surfaces through the application of few nA currents. The following protocol was used: frequency range [100 kHz–10 mHz] with ±10 mV with respect to E_corr_. The polarization resistance value (Rp) was obtained from both LPR and EIS measurements. In particular, the Rp from EIS measurements were calculated as the difference of the modulus |Z| at low and high frequencies [36,37]. A set of E_corr_ measurements were performed on the bulk alloy of the iron fragments of the *San Carlone*. In these cases, the measurements were performed on the polished cross-section 24 h after polishing.

## 3. Results and Discussion

### 3.1. Iron Bulk Alloy Composition

The composition of the subsurface layers was analysed by GDOES (Table 2). Data show that all the samples have a low to very low content of carbon. Sample 46 is almost carbon-free (<0.1%), similar to reference sample A, while in Sample 44, the content of carbon is ~0.25 by weight.

The literature reports that a phosphorous (P) content between 0.1% and 0.6% could be associated with a higher resistance of wrought iron to corrosion [13,23,38,39]. However, the analysis of the samples of the *San Carlone* did not show a relevant presence of phosphorous. In contrast, for the reference sample of puddled iron, a phosphorus content of 0.3% was detected. The composition of Sample 87, the heavily corroded one, is not reported in Table 2, as its dimensions were too small to perform GDOES analysis. The elemental composition has been investigated by SEM-EDX on a polished cross-section, revealing only the presence of Fe, C, and O.

**Figure 4:**
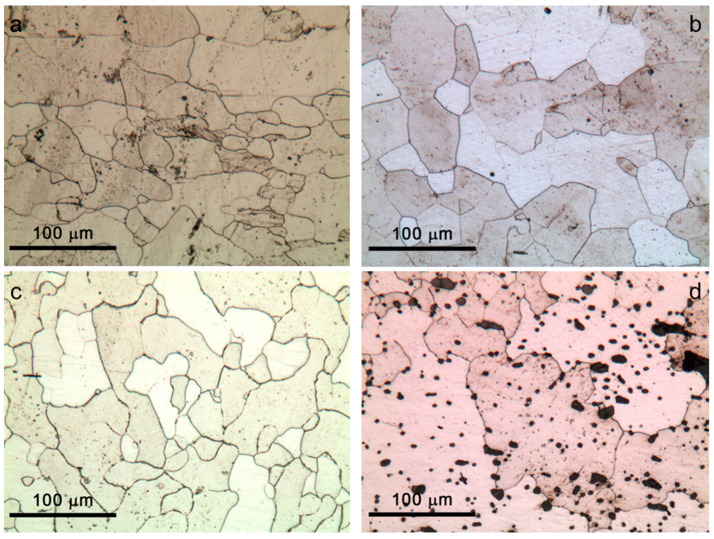
Microstructure observed by optical microscopy in the cross-sections of iron samples from the *San Carlone* and on the reference sample of puddled iron after etching with Nital2. Images of the Sample core: (**a**) Sample 44—slightly corroded sample in contact with copper; (**b**) Sample 46—slightly corroded sample not in contact with copper; (**c**) Sample 87—heavily corroded sample in contact with copper; and (**d**) Sample A—reference sample of puddled iron.

**Figure 5:**
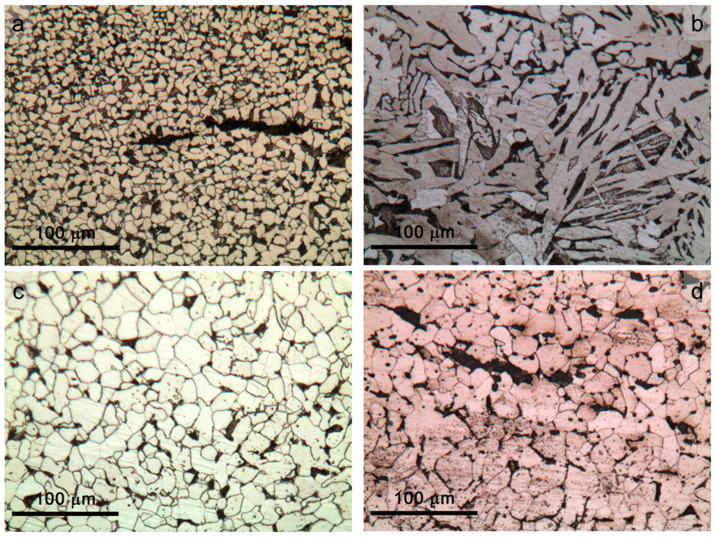
Microstructure observed by optical microscopy in the cross-sections of the iron samples from the *San Carlone* and on the reference sample of puddled iron after etching with Nital2. Outermost areas of the samples: (**a**) Sample 44—slightly corroded sample in contact with copper; (**b**) Sample 46—slightly corroded sample not in contact with copper; (**c**) Sample 87—heavily corroded sample in contact with copper; and (**d**) Sample A—reference sample of puddled iron.

In Figure 4 and Figure 5, images of the microstructures observed in a cross-section of iron samples from the Colossus are reported (bulk in Figure 4, outermost zones in Figure 5). It has been observed that different areas of the samples presented slightly different carbon content and different microstructures, as could be expected for historical iron samples [13,40,41]. Moreover, the predominance of ferritic structure can be observed. In general, the core of the samples from the Colossus showed a ferritic microstructure with coarse grains ranging between about 10 and 100 μm (Figure 4a–c). However, the microstructure of wrought iron samples was not homogeneous. Ferritic–pearlitic microstructures were observed (Figure 5) in discontinuous areas near the surface or at the outer edge of the samples. Moreover, other microstructural heterogeneities were observed across the surface of the polished samples. In Samples 44 and 87 (Figure 5a,c), the ferrite grains showed a rounded shape, and their dimensions became smaller (up to 10 μm) on going from the bulk towards the surface. In Sample 46, the ferrite grains in ferritic–pearlitic areas just below the surface showed a Widmanstätten structure (Figure 5b) [42] with the exception of the most superficial area (about a couple of μm thick) where ferrite with small and round-shaped grains became predominant again. In hypoeutectoid steels such as those investigated here, Widmanstätten structures may form mainly through rapid cooling combined with coarse prior austenitic grain size (leading to an insufficient number of nuclei for conventional proeutectoid ferrite crystallization) [42,43,44]. The abovedescribed microstructural heterogeneity in these samples can be ascribed to surface carburization/decarburization cycles during the hot forging process, typical of wrought iron produced in the reference period for this statue [40]. The Widmanstätten structures detected in bands parallel to the outer surface are probably due to localised fast cooling during the forging cycles. Moreover, the puddled iron reference sample showed a banded structure with the alternation of ferritic areas with grain size ranging from ~100 μm (Figure 4d) to ~10 μm or lower (Figure 5d). In particular, such a microstructure is caused by the intrinsic heterogeneity of this material since the puddling process does not allow full homogenization [39].

**Figure 6:**
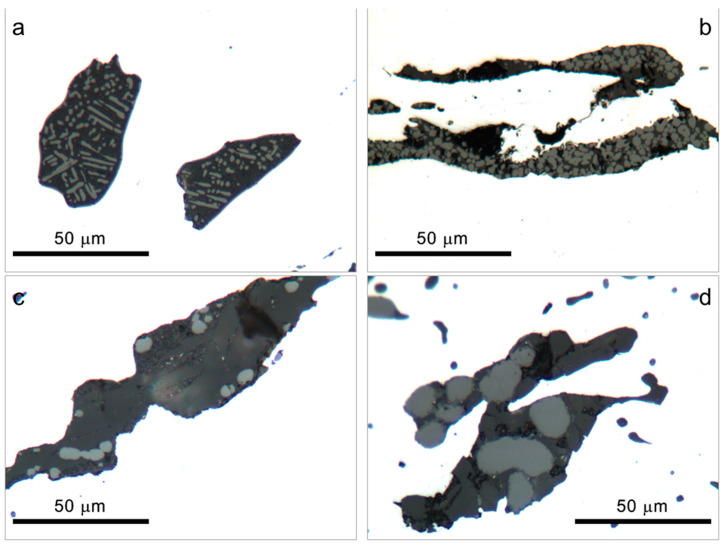
Inclusions observed by optical microscopy on nonetched cross-sections of the *San Carlone* iron samples and the reference sample of puddled iron. (**a**) Sample 44—slightly corroded sample in contact with copper; (**b**) Sample 46—slightly corroded sample not in contact with copper; (**c**) Sample 87—heavily corroded sample in contact with copper; and (**d**) Sample A—reference sample of puddled iron.

For historical wrought iron, the corrosion and mechanical behaviour of the metal is strongly influenced by the amount, dimensions, orientation, and composition of the slag inclusions [13,23,39,45,46]. In general, for historical irons, the presence of a high number of inclusions with strongly variable dimensions is expected [13,40,45]. Inclusions are typically constituted by a glass matrix of fayalite (Fe_2_SiO_4_) with wüstite (FeO) crystals [13,39] and are usually arranged in parallel bands due to forging [39]. Both in samples of *San Carlone* and on the reference A sample (puddled iron), slag inclusions with dimensions ranging from about 50 μm to millimetric dimensions (Figure 6) were present. Moreover, in Sample A, smaller etching pits, typical of phosphorus-rich irons [39], could be observed (Figure 6d). In all the samples, small crystals (light areas) are visible in a glass matrix (darker areas in Figure 6) with the typical fayalite-wüstite structure confirmed by micro-Raman spectroscopy.

The observed good corrosion behaviour of iron elements may be possibly associated with the presence of slag inclusions. As discussed by Chang et al. [47], in the case of historic copper, the dimensions and nobility of inclusions may influence the composition of corrosion products and therefore the protection they provide to the underlying alloy.

### 3.2. Iron Corrosion Products Composition

**Figure 7:**
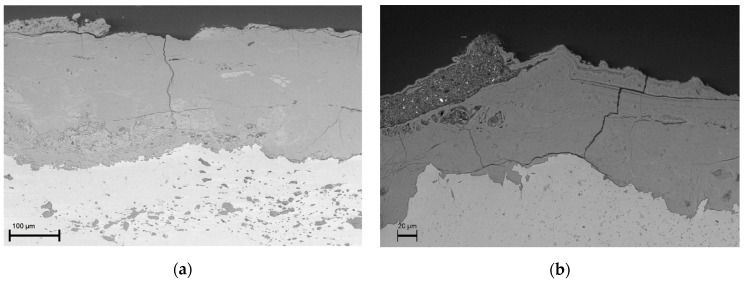
SEM images of Samples 44 (**a**) and 87 (**b**).

**Figure 8:**
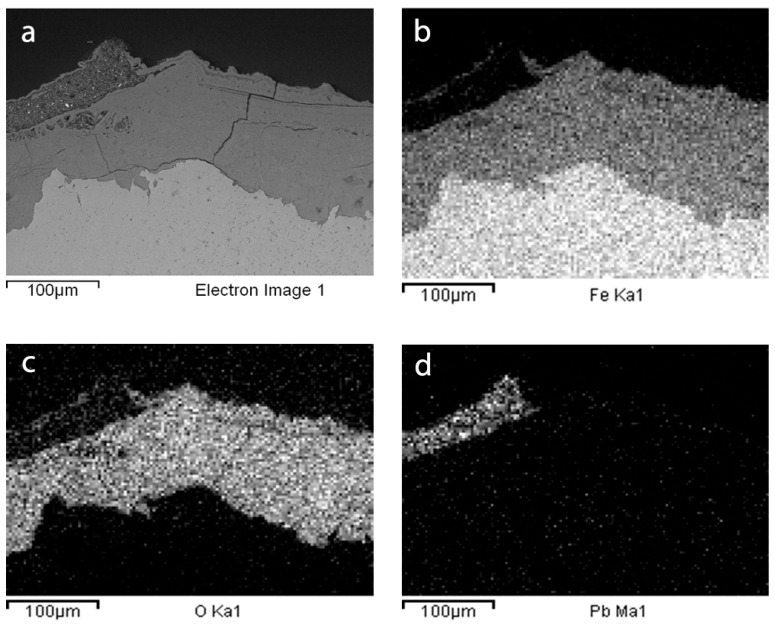
EDX maps of Sample 87 (**a**) Electron Image, (**b**) Iron Ka1, (**c**) Oxigen Ka1, and (**d**) Lead Ma1.

Corrosion products of the areas adjacent to Samples 44 and 46 looked compact and highly adherent to the surface. They were characterised by a dark brown-blackish colour. Sample 87 was a small lamina of iron surrounded by a thick layer (>1 cm) of reddish-brown corrosion products that peel off and disintegrate easily and that detached during sampling. When investigated by SEM/EDX, all samples presented a compact and adherent corrosion layer (Figure 7 and Figure 8). EDS X-ray maps in Figure 8d also revealed some remnants of a Pb-containing antirust paint layer (the so-called “red lead paint” which was frequently used a few decades ago) above the corrosion products on the surface of Sample 87. 

Atmospheric-generated iron corrosion layers are typically constituted by oxides, hydroxides, or oxides–hydroxides [11,12,13]. Depending on their relative abundance, the PAI index can be calculated, thus quantifying the protectiveness of the corrosion layers [14,26]. 

To assess the protective role of the corrosion layers against galvanic corrosion of the *San Carlone*, their corrosion products have been investigated using micro-Raman spectroscopy and XRD. Micro-Raman spectroscopy analysis was performed on polished cross-sections to evaluate the stratigraphy of corrosion products, whilst XRD was performed directly on the surface of the fragments. The results of these analyses are summarised in Table 3.

**Figure 9:**
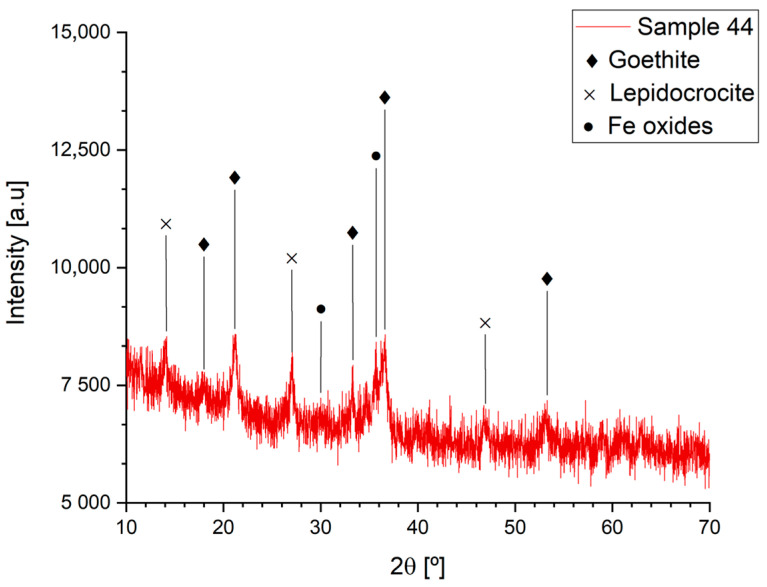
XRD analysis of sample 44.

In all the examined fragments, XRD analysis detected goethite and lepidocrocite as the main corrosion products. The former is typically considered among the protective corrosion products, and the latter is normally more reactive. Moreover, iron oxides have been identified in traces. In contrast, micro-Raman spectroscopy identified a larger number of corrosion products where goethite and lepidocrocite were again the main ones. Goethite and lepidocrocite were found especially in the intermediate layer between the most internal one and the external layer. The internal layer was compact and richer in iron oxides (magnetite, maghemite, and hematite), whereas the composition of the external one was influenced by the presence of environmental contaminants and pollutants. The latter observation could also explain the widespread presence of akaganeite, an iron oxide–hydroxide that usually contains 5–8 wt.% Cl. This data is in good accordance with the quite diffused presence of atacamite observed among the copper corrosion products. There might be three possible explanations for the presence of chlorides in Arona: They could have been transported by the wind to the Colossus; they could have been present in the past in the atmosphere as pollutants; or they could derive from the use of chloride-containing cleaning product during past restoration interventions. In addition, hematite has been detected in Sample 46. However, it should be considered that its presence could be partly due to a transformation of the corrosion products upon heating of the sample by the laser during Raman analysis. Moreover, a high fraction of amorphous phases has been highlighted by XRD analysis (Figure 9). For this reason, a quantification of the relative amount of each phase was impossible, as well as the evaluation of the protectiveness of corrosion layers based on the PAI index. Apparently, therefore, no significant differences can be observed in the chemical composition of the corrosion layers that could explain the different corrosion behaviour of the samples.

The same analysis was also performed on powder samples of corrosion products collected from iron bars with evidence of active corrosion (not necessarily in direct contact with copper sheets, which provided similar results. Both XRD and micro-Raman analyses identified goethite and lepidocrocite as the main corrosion products in association with magnetite, maghemite, and hematite. On powder samples akaganeite was also detected. Moreover, for these samples, a lower variety of corrosion products were detected by XRD than by micro-Raman, basically identifying goethite, lepidocrocite, and a low amount of hematite on a few samples. Moreover, on powder samples, significant amounts of deposits and contaminants were detected. In particular, gypsum was typically identified in the areas with evident active corrosion. Gypsum normally reaches the surfaces by wet and dry deposition, thus explaining the high amounts of deposits on the surface. Furthermore, the presence of gypsum could suggest that the higher corrosion rate of those iron bars might be associated with specific microclimatic conditions promoted by deposition or condensation phenomena. In fact, the presence of more severe corrosion phenomena in association with high amounts of deposits and condensation was already observed during the preliminary inspections of the monument prior to the restoration of 1974–1975 [2]. 

### 3.3. Interaction of Copper and Iron Elements

The electrochemical behaviour of the selected copper and iron elements has been investigated. The aim was to understand the good conservation state of the iron elements from the corrosion point of view and to explain the scarce presence of galvanic corrosion between copper and iron. In particular, corrosion potential (E_corr_) measurements and polarization resistance (Rp) measurements were performed.

**Figure 10:**
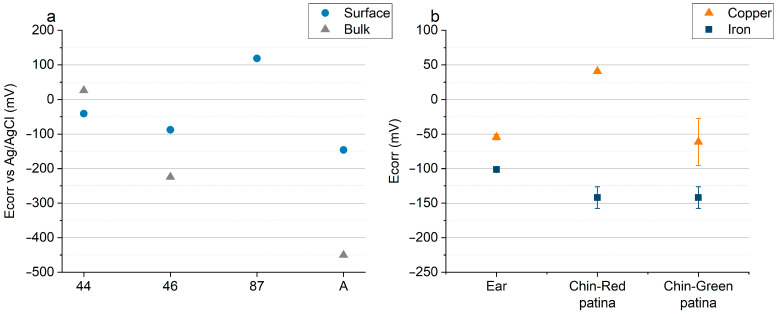
Corrosion potential (E_corr_) measured on surfaces of the *San Carlone* with a Ag/AgCl reference electrode. (**a**) E_corr_ measurements on the surfaces and the polished cross-sections of iron fragments; (**b**) on-site E_corr_ measurements on adjacent copper and iron surfaces.

**Figure 11:**
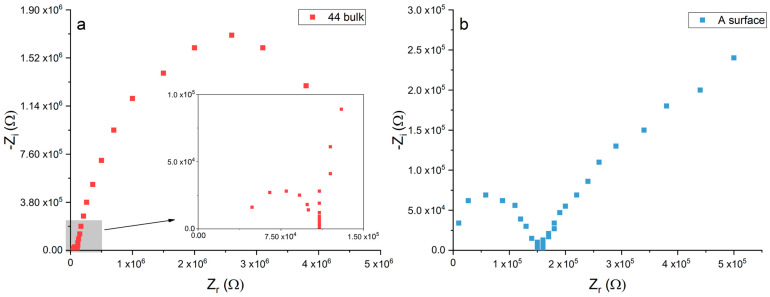
Nyquist plots of Sample 44 (**a**) and Sample A (**b**).

**Figure 12:**
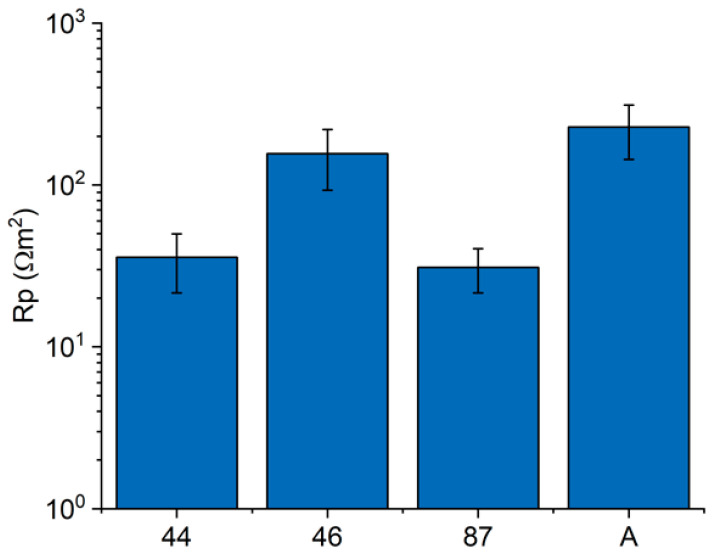
Polarization resistance measurements on iron fragments from the *San Carlone* of Arona.

Figure 10a shows the E_corr_ values measured in the laboratory on the surfaces and polished cross-sections of the iron samples from the Colossus and on the reference sample A. On Sample 87, only surface measurements could be performed due to its very small dimensions. The bulk of Samples 44 and 46 show a more noble potential (25 mV and −225 mV vs. Ag/AgCl) with a E of +475 mV and +225 mV, respectively, with respect to the puddled iron (E_corr_ = −450 mV vs. Ag/AgCl). Moreover, the corrosion potentials measured on the surfaces of such samples are quite noble. Surprisingly, the highest value (+120 mV vs. Ag/AgCl) has been measured on the surface of the most corroded sample (87). Interestingly, the difference in corrosion potential between the surface and bulk alloy of Samples 44 and 46 is very low and significantly lower than the one measured on the reference sample A. Figure 10b shows the results of on-site E_corr_ measurements in the statue on adjacent copper and iron surfaces. In this case, all the iron bars appeared slightly corroded but were covered by compact corrosion layers adherent to the surface. The potential difference between the two surfaces never exceeded 200 mV, and in two cases out of three, it was lower than 100 mV. The three iron fragments from the Colossus and the reference sample A were electrochemically characterized by measuring the polarization resistance of their surfaces with LPR and EIS. Nyquist plots of Sample A (surface) and Sample 44 (section) are reported in Figure 11a,b, respectively. Average Rp values (Figure 12) were higher than 30 Ω·m^2^. The surfaces of Samples 44 and 87 resulted in the least resistance to corrosion. Sample 46 and the reference sample A showed Rp values higher than 150 Ω·m^2^, suggesting a good corrosion resistance.

**Figure 13:**
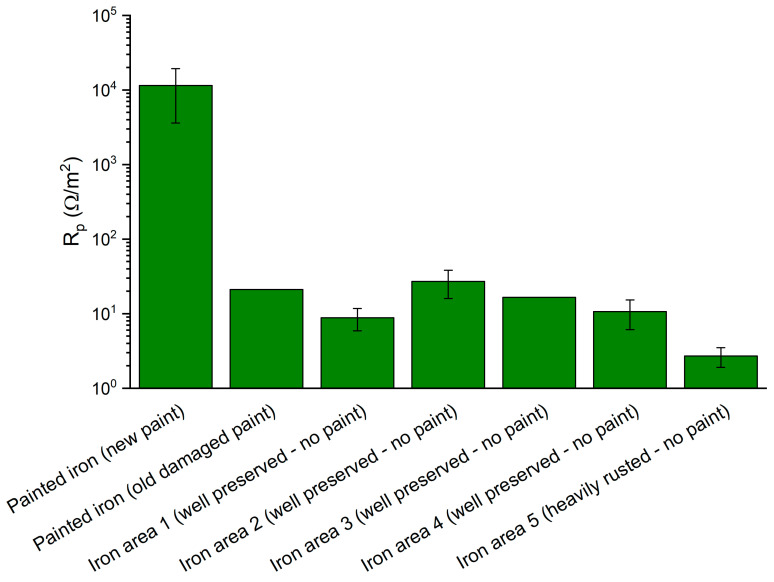
In situ polarization resistance measurements on iron elements of the *San Carlone* of Arona.

The Rp measurements, performed in situ, of different iron elements of the *San Carlone*, characterised by different conservation conditions are displayed in Figure 13. Only a few areas were characterised by the presence of a protective coating (“painted iron-new paint”), while most areas were characterised by a fairly good conservation condition despite the lack of paint (“well preserved”). These “well preserved” areas showed Rp measurements similar to the ones obtained from the fragments analysed in the laboratory, suggesting a relatively low corrosion rate. Rusted areas (“heavily rusted”) were those showing the lowest polarisation resistance.

These results suggest that the iron alloy used for the construction of the Colossus generally presents a noble open circuit potential. Indeed, its corrosion potential resulted few tens of mV lower than the one of copper surfaces. Therefore, it could be hypothesized that the free corrosion potential difference between copper and iron was low for the development of an effective galvanic coupling.

Recently, oxygen depletion measurements [48] allowed for the classification of iron objects cared for by the English Heritage into four categories of corrosion behaviour. Surprisingly, a high number of objects were classified into Category 1 or 2. Particularly, Category 1 is related to a material that does not appear to deteriorate even up to very high RH values. Some sites reached 85% RH, but no sign of deterioration has been observed visually over 20 years of exposure. These observations are confirmed by oxygen testing of representative samples that show no detectable deterioration at 75% RH. No clear explanation of the good corrosion behaviour of such iron objects has yet been identified. 

Unfortunately, due to the millimetric size of San Carlone samples, it was not possible to perform oxygen depletion measurements. However, the description of Category 1 objects closely resembles the observation of most areas of iron elements in the San Carlone. As previously discussed, a clear correlation between alloy composition and conservation condition could not be identified. Data and observations suggest that good corrosion resistance may be mainly ascribed to the presence of slag inclusions and favourable environmental conditions. Further investigations are required to provide a deeper understanding of this phenomena. 

## 4. Conclusions

Both the bulk and the surface of the iron elements of the monumental statue of *San Carlone* of Arona showed quite good corrosion resistance. Galvanic corrosion is not expected to be relevant since their free corrosion potential was only 200 mV (or less) lower than that of the copper surfaces. The reason for the rather noble corrosion potential of iron elements is still unclear. 

The micro-Raman and XRD analyses of the corrosion products did not highlight any significant difference in the chemical composition of the corrosion products among different samples. Moreover, the investigation of the microstructure of the iron samples did not allow for an explanation as to why active corrosion was observed only in a few limited areas. The analysed sample was characterised by great heterogeneity of the iron-based material, consisting of three main microconstituents: ferrite, pearlite, and multiphase slag. The relatively noble corrosion potential and good corrosion behaviour of iron elements seem to be associated both with favourable environmental conditions (low chlorine content and low pollution) and possibly with the presence of slag inclusions with dimensions ranging from about 50 μm to ~1 mm.

The obtained results allow us to hypothesise that the corrosion phenomena observed only in a few areas may be promoted by specific and localized microclimatic conditions, associated with deposits and condensation phenomena. This hypothesis could be supported by the significant presence in the corroded areas of gypsum, soil, and dust deposits, which may enhance corrosion due to their hygroscopicity. 

To corroborate the obtained results and better understand the good corrosion behaviour of the *San Carlone* iron alloys, it would be interesting to analyse other similar case studies with comparable environmental conditions and to perform further laboratory testing with different historical wrought irons.

## Figures and Tables

**Table 1:** Average pollutants concentrations: data obtained from the monitoring station near Arona (from the regional environmental protection agency (ARPA) website).

**Borgomanero-Molli**						
**Pollutant**	**Year**	**Units**	**Average**	**Min**	**Max**	**Sd**
NO_2_	2018	µg·m^−2^	24.4	1.0	115.0	16.0
	2019	µg·m^−2^	29.0	1.0	129.0	19.2
NO	2018	µg·m^−2^	12.7	0.0	245.0	21.4
	2019	µg·m^−2^	12.1	0.0	483.0	21.3
NO_x_	2018	µg·m^-2^	43.7	2.0	474.0	45.9
	2019	µg·m^−2^	47.3	2.0	773.0	48.3
CO	2018	mg·m^-2^	0.4	0.1	2.5	0.3
	2019	mg·m^−2^	0.5	0.1	1.9	0.3
PM2.5	2018	µg·m^−2^	14.8	1.0	66.0	10.4
	2019	µg·m^−2^	14.9	1.0	73.0	10.3
PM10	2018	µg·m^−2^	17.8	1.0	80.0	11.6
	2019	µg·m^−2^	16.9	1.0	87.0	11.4
**Castelletto Sopra Ticino-Fontane**						
**Pollutant**	**Year**	**Units**	**Average**	**Min**	**Max**	**Sd**
NO_2_	2018	µg·m^−2^	21.6	2.0	86.0	13.2
	2019	µg·m^−2^	22.6	3.0	94.0	13.7
NO	2018	µg·m^−2^	11.3	0.0	155.0	18.6
	2019	µg·m^−2^	10.5	1.0	192.0	15.8
NO_x_	2018	µg·m^−2^	38.9	2.0	294.0	37.1
	2019	µg·m^−2^	38.6	8.0	338.0	32.5
PM10	2018	µg·m^−2^	19.8	1.0	70.0	12.6
	2019	µg·m^−2^	-	-	-	-
O_3_	2018	µg·m^−2^	45.1	1.0	199.0	40.1
	2019	µg·m^−2^	38.6	8.0	338.0	32.5

**Table 2:** Bulk alloy composition of iron samples (wt.%) obtained by GDOES measurements.

Code	Sample	Fe	C	Mn	Si	P	S	Cr	Ni	Cu	Co
44	Slightly Corroded—Contact with Cu	99.2	0.26	0.01	0.004	0.03	0.01	0.001	0.003	0.40	0.03
46	Slightly corroded—No contact with Cu	99.9	0.009	0.02	–	0.04	0.007	0.001	0.009	–	0.04
A	Reference puddled iron	99.3	0.006	0.05	0.10	0.30	0.01	0.02	0.04	0.01	0.04

**Table 3:** Corrosion products detected by Raman spectroscopy and XRD on the *San Carlone* samples.

Sample	Layer	Raman	XRD
44	Metal–rust interface	Goethite (αFeOOH) Magnetite (Fe_3_O_4_) Maghemite (γ-Fe_2_O_3_)	Goethite (αFeOOH) Lepidocrocite (γFeOOH) iron oxide in trace
	Intermediate layer	Goethite (αFeOOH) Lepidocrocite (γFeOOH)	
	External layer	Maghemite (γ-Fe_2_O_3_) Akaganeite (βFeOOH)	
46	Metal–rust interface	Goethite (αFeOOH) Lepidocrocite (γFeOOH) Akaganeite (βFeOOH)	Goethite (αFeOOH) Lepidocrocite (γFeOOH)
	Intermediate layer	Goethite (αFeOOH) Maghemite (γ-Fe_2_O_3_)	
	External layer	Hematite (α-Fe_2_O_3_) Lepidocrocite (γFeOOH) Akaganeite (βFeOOH)	
87	Metal–rust interface	Goethite (αFeOOH)	Goethite (αFeOOH) Lepidocrocite (γFeOOH) Iron oxide (traces)
	Intermediate layer	Goethite (αFeOOH)	
	External layer	Goethite (αFeOOH) Lepidocrocite (γFeOOH)	

## Data Availability

The data that support the findings of this study are available from the coordinator of the project, Sara Goidanich (sara.goidanich@polimi.it), upon reasonable request.
